# Body mass index, adjuvant chemotherapy, toxicity, and survival in non-metastatic colorectal cancer: an individual participant data meta-analysis (OCTOPUS)

**DOI:** 10.1038/s41416-026-03472-4

**Published:** 2026-06-09

**Authors:** Corinna G. V. Slawinski, Lee Malcomson, Jorge Barriuso, Hui Guo, Richard Emsley, Richard D. Riley, Andrea Harkin, Timothy Iveson, Robert Glynne-Jones, Cornelis J. H. van de Velde, Andrew G. Renehan

**Affiliations:** 1 Division of Cancer Sciences, School of Medical Sciences, Faculty of Biology, Medicine and Health, Manchester Academic Health Science Centre, University of Manchester, Manchester, UK; 2 Department of Colorectal Surgery, Lancashire Teaching Hospitals NHS Foundation Trust, Preston, UK; 3 Centre for Biostatistics, School of Health Sciences, Faculty of Biology, Medicine and Health, Manchester Academic Health Science Centre, University of Manchester, Manchester, UK; 4 Department of Biostatistics and Health Informatics, Institute of Psychiatry, Psychology & Neuroscience, King’s College London, London, UK; 5 Centre Institute of Applied Health Research, College of Medical Dental Sciences, University of Birmingham, Birmingham, UK; 6 CRUK Glasgow CTU, University of Glasgow, Glasgow, UK; 7 University Hospital Southampton NHS Foundation Trust, Southampton, UK; 8 Department of medical oncology, Mount Vernon Cancer Centre for Cancer Treatment, Northwood, UK; 9 Department of surgery, Leiden University Medical Centre, Leiden, The Netherlands; 10 Colorectal and Peritoneal Oncology Centre, Christie NHS Foundation Trust, Manchester, UK

**Keywords:** Cancer epidemiology, Gastrointestinal cancer, Obesity

## Abstract

**Introduction:**

We aimed to disentangle whether elevated body mass index (BMI) is directly associated with adverse survival in primary colorectal cancer (CRC), or indirectly through treatment-related mechanisms, e.g., dose-capping adjuvant chemotherapy (ACT) and toxicity, using individual participant data meta-analysis (IPD-MA) and causal inference approaches.

**Methods:**

Using BMI from four CRC-ACT randomised trials [OCTOPUS], and two ACT adherence measures (average cumulative relative dose [ACRD]; average relative dose intensity [ARDI]), we performed two-stage random effects IPD-MA, assessing total (TE) and direct effects (DE) (excluding and including mediators, respectively) of pre-defined causal paths, with overall survival (OS) as primary outcome.

**Findings:**

In 7264 patients, the TE of 5 kg/m^2^ BMI increments was a significant ACRD reduction (−1.15% [95% CI −1.92, −0.38]), and ACRD 5% increments were associated with improved OS (HR 0.94 [0.91, 0.96]), implying possible adverse indirect effects; though not large enough to induce an adverse TE of BMI on OS (HR 0.98 [0.90, 1.07]). BMI-ARDI relationships were similar (TE −1.08% [−1.44, −0.72]), but ARDI-OS relationships were inverted (HR 1.05 [1.01, 1.09]). BMI showed no association with grade 3+ toxicity (OR 1.01 (0.91, 1.14)). However, toxicity was associated with worse OS (TE HR 1.37 [1.17, 1.61]), which attenuated on adjusting for ACRD (DE HR 1.20 [1.02, 1.41]), suggesting partial mediation via a significant toxicity-ACRD relationship (−10.37% [−11.77, −8.97]).

**Conclusion:**

Our study establishes possible adverse indirect effects of obesity on CRC survival, through treatment-selection, supporting full BSA-based ACT dosing.

## Introduction

Excess adiposity, expressed as elevated body mass index (BMI), is an established risk factor for 13 cancer types, including colorectal cancer (CRC) [1]. BMI-CRC survival relationships are less clear. Three meta-analyses demonstrate possible adverse relationships between elevated BMI and CRC survival [2–4]. However, some evidence of an obesity paradox also exists [5, 6]. More recently, the World Cancer Research Fund/American Institute for Cancer Research (WCRF/AICR) Global Cancer Update Project published an updated meta-analysis exploring post-diagnosis BMI and colorectal cancer survival outcome relationships. A reverse J-shaped association demonstrated an increased risk of adverse outcome with BMI 18–24 kg/m^2^ and for BMI 32–38 kg/m^2^ [7]. We have previously explored the challenges and limitations to interpreting BMI-survivorship evidence, identifying that poorer outcomes might be indirectly mediated though down-stream pathways such as treatment-related effects, which are rarely accounted for [8]. One important aspect is how dose capping adjuvant chemotherapy (ACT) and toxicity might influence colorectal cancer survivorship.

ACT is dosed according to a patient’s body surface area (BSA), and historically capped at a BSA of 2m^2^ or 2.2m^2^ due to toxicity concerns. Within adjuvant and metastatic CRC, there is some evidence that dose-capping incidence increases with elevated BMI, and outcomes may be improved in fully-dosed vs. dose-capped obese individuals [9–11]. Similar relationships within breast cancer studies have additionally demonstrated associations between elevated BMI and reduced relative dose intensity (RDI), which in turn may reduce survival [12]. Though the dose-capping rationale is based on toxicity concerns, evidence exists for equivalent or indeed reduced toxicity with elevated BMI in fully-BSA-dosed individuals [12].

BMI-dosing-toxicity-survival relationships are complex and incompletely characterised in CRC. Furthermore, no studies have undertaken a detailed analysis of potential causal paths. Hence, whether BMI influences survival through sub-optimal ACT dosing (indirect effects) or directly through other mechanisms (direct effects) remains unclear. We aimed to understand these complex BMI, ACT dosing and toxicity inter-relationships and their effects on survival in non-metastatic CRC, and establish whether BMI might adversely influence survival via indirect treatment-effects of reduced chemotherapy dosing and adherence.

## Methods

Our methodological aims were to explore these relationships, in a setting with reduced biases compared with ‘real world’ data. Our hypotheses were not dealing with treatment comparisons of specific chemotherapy regimens or surgical approaches, hence, we employed a degree of pragmatism for the data acquisition process. However, we employed IPD-MA principles as far as possible, through implementation of Preferred Reporting Items for a Systematic Review and Meta-analysis of Individual Participant Data (PRISMA-IPD) guidance [13]. We included four ACT trials for non-metastatic CRC with available BMI and cycle-level chemotherapy dosing and toxicity data from the OCTOPUS consortium [14], a collection of colorectal and endometrial cancer surgical and ACT randomised trials with IPD, established to examine BMI-survival relationships. The trials included MOSAIC [15, 16], SCOT [17], CHRONICLE [18], and PROCTORSCRIPT [19]. MOSAIC trial data were obtained through Project Data Sphere, with only the control arm data available. Data from SCOT, CHRONICLE and PROCTORSCRIPT were shared from the clinical trial units following data transfer agreements. A detailed data checking, cleaning and harmonisation process was undertaken, also assessing data quality and potential bias using the relevant domains of the Cochrane Risk of Bias 2 (RoB2), which we modified by extending assessment to include biases in relation to exposure, mediator and confounders in addition to outcomes (appendix, supplementary item [SI] 1) [20].

Inclusion criteria were: derivable BMI at trial entry, randomisation to an ACT arm, and receipt of one chemotherapy cycle minimum. Hence, the observation arms of CHRONICLE and PROCTORSCRIPT, and 55 patients from SCOT who withdrew pre-randomisation were excluded. Exclusion criteria were: individuals with missing or extreme BMI (<15 or ≥60 kg/m^2^), metastases, missing outcomes or covariates (as missing data was low), except for those with missing toxicity data from the SCOT trial (as this was high), in addition to those with unlikely chemotherapy adherence measures (appendix, SI 2). We treated SCOT as two discrete populations according to randomisation arm (3 or 6 months). Ethical approval was not required, however, this study formed part of the OCTOPUS project registered with PROSPERO [14].

The primary exposure was BMI [weight (kg)/height (m^2^)], calculated at trial entry. We calculated body surface area using the Mosteller formula [BSA (m^2^) = sqrt((height [cm] × wight [kg])/3600)], as this was the most commonly cited method in the dose-capping literature [12, 21–24]. Chemotherapy dosing calculations (detailed in appendix SI 3 and 4) were performed using actual body weight based BSA, hence, total received doses were divided by each individual’s BSA to obtain doses in mg/m^2^. We calculated cycle one relative dose received (RDR: actual cycle dose [mg/m^2^]/expected cycle dose [mg/m^2^] * 100). We defined dose capping as receipt of <95% of the standard dose (i.e., <95% RDR) of any drug for cycle one, consistent with previous studies [10, 23, 25]. Two summary measures of adherence were calculated for each drug across the entire chemotherapy regimen, the cumulative relative dose (CRD) and relative dose intensity (RDI):

$${{\rm{CRD}}}= 	({{\rm{actual}}}\; {{\rm{cumulative}}}\; {{\rm{dose}}}[{{\rm{mg}}}/{{{\rm{m}}}}^{2}]/{{\rm{expected}}}\; {{\rm{cumulative}}}\; \\ 	{{\rm{dose}}}[{{\rm{mg}}}/{{{\rm{m}}}}^{2}])* 100$$

$${{\rm{RDI}}}= 	({{\rm{actual}}}\; {{\rm{dose}}}\; {{\rm{intensity}}}[{{\rm{mg}}}/{{{\rm{m}}}}^{2}/{{\rm{week}}}]/{{\rm{expected}}}\; {{\rm{dose}}}\; \\ 	{{\rm{intensity}}}[{{\rm{mg}}}/{{{\rm{m}}}}^{2}/{{\rm{week}}}])* 100$$

Where dose intensity is the cumulative dose divided by treatment duration in weeks. The RDR, CRD and RDI were averaged across the drugs in the regimen to obtain the average relative dose received (ARDR), average cumulative relative dose (ACRD) and average relative dose intensity (ARDI). Expected chemotherapy doses and durations were those from the trial protocols and/or published papers (appendix, SI 3). The decision to explore ACRD in addition to ARDI was due to the concern that ARDI would not adequately capture early treatment discontinuation. For example, a fully BSA-dosed patient discontinuing treatment after only one of six cycles would have an ARDI of 100% (ACRD 16.6%), as would a fully BSA-dosed patient receiving all six cycles (ACRD 100%). We assessed the occurrence of any ≥ grade 3 (G3+) toxicity event, defined according to the respective trials.

The primary outcome was overall survival (OS), defined as time from randomisation to death from any cause. Secondary survival outcomes were disease-free survival (DFS), defined as loco-regional or metastatic disease recurrence, a new CRC primary tumour or death from any cause, and cancer-specific-survival (CSS), defined as death from CRC. Intermediate outcomes were dose capping, ARDR, ACRD, ARDI, and G3+ toxicity.

**Fig. 1:**
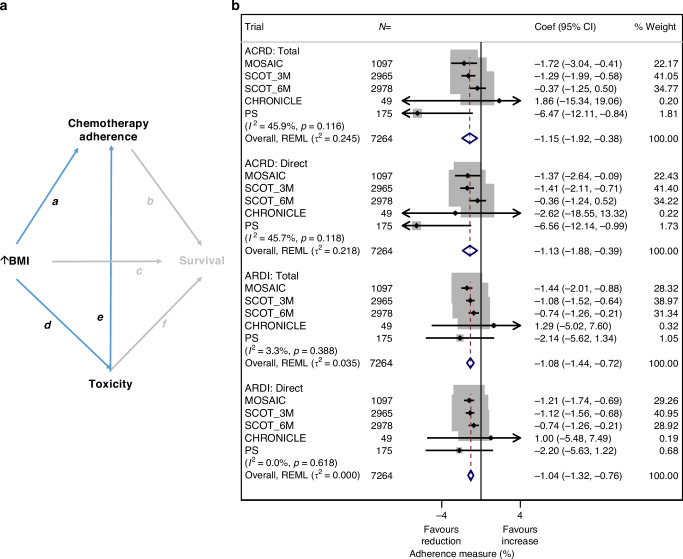
Path *a.* **a** DAG demonstrating the hypothesised causal pathway between BMI and chemotherapy adherence, mediated via toxicity. **b** Forest plot demonstrating the total (not adjusting for toxicity) and direct (adjusting for toxicity) effects of 5 kg/m^2^ increments of BMI on ACRD and ARDI.

**Fig. 2:**
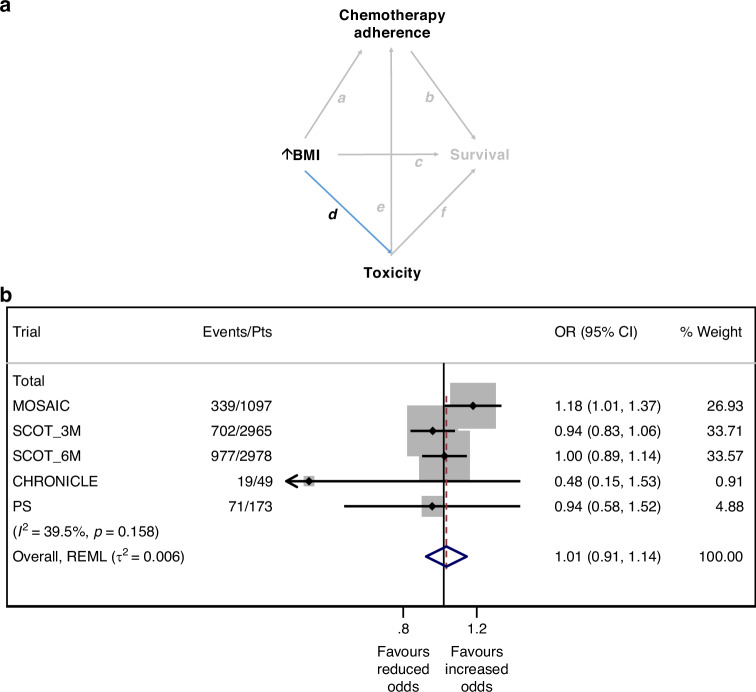
Path *d.* **a** DAG demonstrated hypothesised causal association between BMI and Toxicity. **b** Forest plot demonstrating the effect of 5 kg/m^2^ increments of BMI on the odds of developing grade 3+ toxicity.

**Fig. 3:**
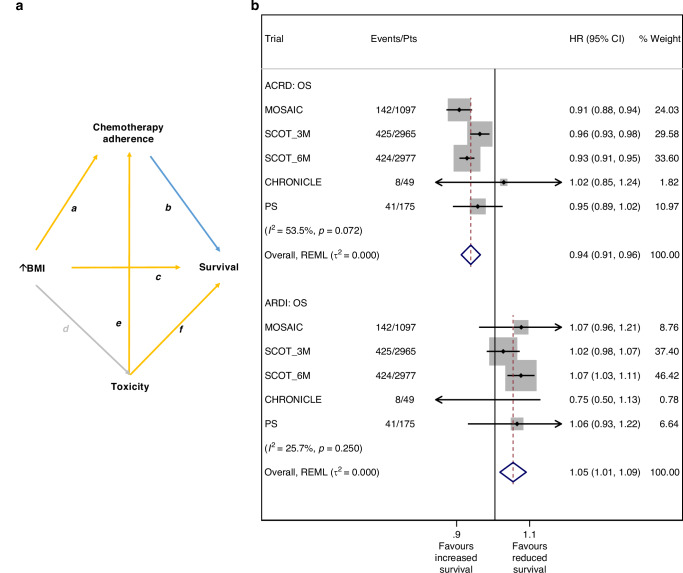
Path *b.* **a** DAG demonstrating hypothesised causal association between chemotherapy adherence and survival. **b** Forest plot demonstrating the effect of 5% increments of ACRD and ARDI on overall survival (OS).

**Fig. 4:**
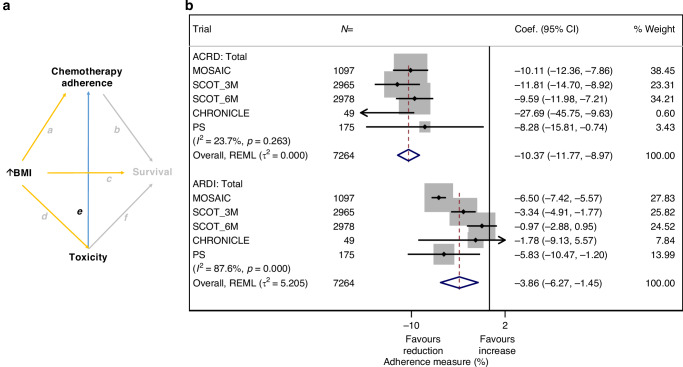
Path *e.* **a** DAG demonstrating hypothesised causal association between Grade 3+ toxicity and chemotherapy adherence. **b** Forest plot for the effect of Grade 3+ on ACRD and ARDI.

**Fig. 5:**
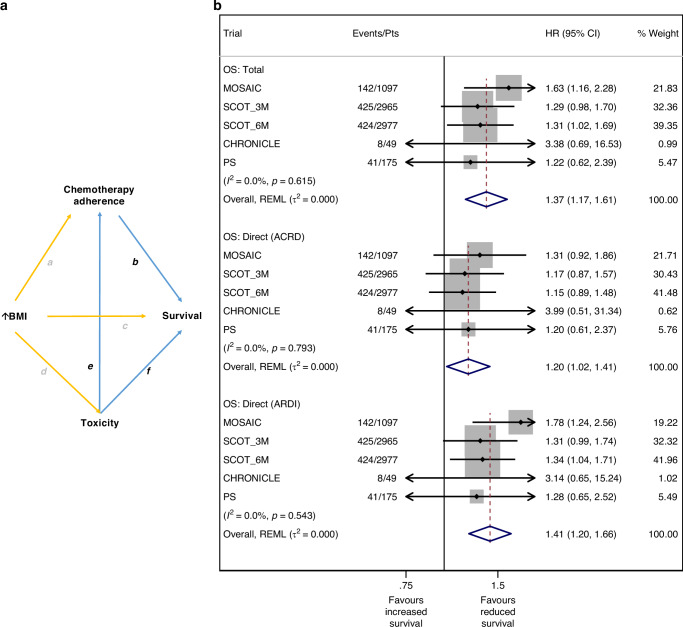
Path *f.* **a** DAG demonstrating hypothesised causal association between Grade 3+ toxicity and survival, mediated by chemotherapy adherence. **b** The total (not adjusted for ACRD/ARDI) and direct (adjusted for ACRD/ARDI) effect of Grade 3+ on overall survival (OS).

**Fig. 6:**
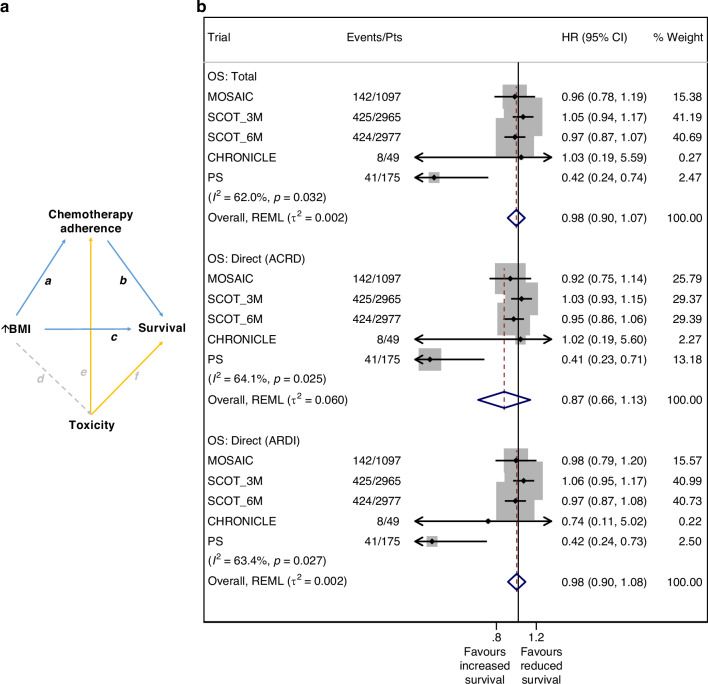
Path *c.* **a** DAG demonstrating hypothesised causal association between BMI and survival, mediated through chemotherapy adherence, with toxicity as a confounder of the mediator-outcome relationship. **b** The total (not adjusted for ACRD/ARDI) and direct (adjusted for ACRD/ARDI) effects of 5 kg/m^2^ BMI increments on overall survival (OS).

We summarised trial-level characteristics with median and interquartile range (IQR) for continuous measurements or frequency (N) and percentages for categorical variables. A causal inference approach was adopted to predefine putative causal paths, analyses, confounders, and specify assumptions. We created directed acyclic graphs (DAGs) for the overall relationship between BMI, ACT adherence, G3+ toxicity and survival (appendix, SI 5), in addition to individual paths (Figs. 1a–6a). We hypothesised that BMI is causally associated with survival (path *c*) and partly mediated indirectly by ACT adherence (i.e., via BMI-adherence [path *a*; due to dose capping] and adherence-survival [path *b*] relationships). Furthermore, we theorised that BMI might be associated with G3+ toxicity [path *d*], and in turn associated with survival [path *f*], the latter both directly, and indirectly via reduced ACT adherence [path e] (due to dose modifications, delays, and early discontinuation).

We performed two-stage random-effects univariate IPD meta-analysis to maintain trial clustering and allow for between-trial heterogeneity. First, fitting trial-level models to obtain parameter estimates and variances using linear, logistic or Weibull survival regression models for continuous, binary, and survival outcomes, respectively. We used Weibull survival models, as VanderWeele [26] has previously demonstrated their ability to provide valid estimates of mediation in comparison to Cox Proportional Hazards models in the context of a common outcome. A sensitivity analysis (data not presented) confirmed comparable results with Weibull and Cox models, suggesting that the Weibull distribution was appropriate to model the baseline hazard. All analyses were adjusted for age, sex, pathological tumour (pT) and nodal (pN) stages, and ECOG performance status, plus BMI and/or toxicity for certain paths. We avoided categorising continuous exposures (e.g., BMI) to prevent information loss, initially assuming linear associations between continuous exposure and outcome. Second, we combined trial-level parameter estimates and variances using the random-effects model, fitted using restricted maximum likelihood estimation (REML), which weights trials according to the inverse of the sum of the estimated trial-specific variances and the between-study variance. Heterogeneity was assessed using Tau^2^, where no heterogeneity results in a value of zero [27, 28].

The SCOT trial contained a high proportion of patients with missing toxicity data due to the pre-defined safety-monitoring protocol [17]. Additional “ad hoc” toxicity data appeared to have been collected for 921 patients; this was hypothesised to result from delays in informing trial centres that toxicity data collection could cease (indeed delay in notification was noted by the study authors). To avoid complete-case analysis bias, we employed multiple imputation (MI) for models including toxicity, assuming data were missing at random. We used the mice.impute.rf package within the mice R package for MI with 10 random forests [29] to iteratively generate 10 imputed datasets, running the regression models on each, and combining the coefficients and standard errors using Rubin’s Rules [30]. Toxicity was imputed at the patient level for the whole treatment regimen. MI predictors included: BMI, age, sex, ECOG performance status, pT stage, pN stage, chemotherapy regimen, ARDI and ACRD, cycle 1 RDR, randomisation date, surgery date, tumour site, baseline BSA, weight and height. Furthermore, we generated two complete-case datasets (TOX1 and TOX2) for sensitivity analysis. Missing data patterns were explored, and importantly, did not appear to be related to grade of reported toxicity. However, missingness increased with time from randomisation in keeping with the above hypothesised mechanism of missingness, hence including randomisation date as an MI predictor.

The DAGs demonstrate the putative mediated paths (SI 5 and Figs. 1a–6a). For mediation to exist, there must be an exposure-mediator, and mediator-outcome relationship [31]. The traditional difference of coefficients mediation analysis approach involves fitting two regression models, first without the mediator (total effect of exposure on outcome) and second with the mediator (direct effect of exposure on outcome). The indirect effect (via the mediator) can be obtained by subtracting the direct effect from the total effect [31]. Though this method cannot generate indirect effect standard errors, and thus cannot be meta-analysed, the magnitude and direction of change in effect estimate can give an indication of potential mediation. Thus, we explored putative causal paths and modelled total (excluding the mediator) and direct (adjusted for the mediator) effects for mediated paths.

We assessed for non-linearity of continuous exposures’ association with outcomes, using restricted cubic splines. Multivariate REML random-effects meta-analysis was performed using correlated spline term effects, their variances and co-variances, graphing predicted outcome values against the exposure to assess for non-linearity [32]. Following sensitivity analysis of knot placement (including 3-, 4- and 5-knot models), which all yielded similar shaped curves, the three-knot model was found to be the best-fitting model. Hence, this was utilised, defining the knots in the combined IPD data (i.e. fixed at the same absolute values of BMI 20 kg/m^2^, 25 kg/m^2^ and 30 kg/m^2^ for each study) [33].

We explored interaction effects for sex and disease stage (utilising SCOT definitions of low and high risk stage III disease [17]). Finally, we performed sensitivity analyses to explore effects of: 6 months of treatment (excluding SCOT 3 month arm); dose banding (excluding patients receiving CAPOX/capecitabine); reverse causality (exclusion of deaths within 6 months of randomisation); possible data errors; missing data (complete case analysis); small trial effects (exclusion of CHRONICLE and PS); and more conservative 95% confidence interval estimates using the Hartung-Knapp-Sidik-Jonkman (HKSJ) method. Results are reported according to PRISMA-IPD guidelines.

Statistical analyses were performed in Stata version 17 (StataCorp LLC, 2017, College Station, TX, USA), and imputed datasets were generated in R version 3.6.2. and R Studio (version 1.4.1106). Data could not be made available, as this does not belong to the study authors, but rather the individual trials, consequently Stata and R code have not been made available.

## Results

Following exclusions, a total of 7264 patients were included (appendix, SI 2). Trial baseline characteristics are displayed in Appendix SI 6. Both SCOT trial arms had a higher median BMI (higher proportion of obese patients), representative of more modern populations, with patients tending to be older. WHO performance status tended to be higher in MOSAIC. There were also differences in proportions of female patients, (y)pT and (y)pN and AJCC disease stages across trials.

Risk of bias and data quality assessment are summarised in the appendix (SI 1). Overall, the data were good quality, and concerns over missing toxicity data and other potential minor data errors were addressed through multiple imputation methods and sensitivity analyses.

First, we assumed a linear association of BMI with the outcome. BMI 5 kg/m^2^ increments were significantly associated with almost three times increased odds of dose capping (OR 2.67; 95% CI 1.97, 3.62), equating to −2.11% cycle 1 ARDR (95% CI −2.54, −1.68 [appendix, SI 7]). The BMI-adherence relationship (path *a*) is presented in Fig. 1. The total effect of 5 kg/m^2^ BMI increments was a 1% reduction of both ACRD (−1.15%; 95% CI −1.92, −0.38) and ARDI (−1.08%; 95% CI −1.44, −0.72). Adjusting for toxicity made minimal difference to effect estimates, and no BMI-G3+ toxicity relationship was demonstrated (OR 1.01; 95% CI 0.91, 1.14; path *d*, Fig. 2), suggesting no mediation of path *a* via toxicity. Furthermore, no significant within-trial BMI-dose capping interaction effect was seen on G3+ toxicity occurrence (appendix, SI 8). Finally, within the BMI-dose capping interaction models, meta-analysis of the effect of dose-capping demonstrated a non-significant tendency to reduce toxicity (OR 0.84; 95% CI 0.66, 1.07). Hence, toxicity appeared to be independent of both BMI and of dose capping, with no significant interaction effect, potentially explaining the lack of association between BMI and toxicity despite higher rates of dose capping.

Non-linear associations are demonstrated in Appendix SI 16. There was evidence of non-linearity for BMI-dose capping relationships. The association was flat for BMI up to 25 kg/m^2^, after which the odds increased with a slope steeper than for linear associations, suggesting linear models might slightly under-estimate the reduction in adherence associated with increasing BMI. These associations were mirrored by BMI-ARDR, BMI-ARDI and BMI-ACRD relationships.

We then assessed the downstream effects of adherence on survival, assuming a linear association. There were 1040 (14.3%) deaths occurring during a median of 3.05 (IQR 2.90, 4.00) years follow-up. Adherence-survival relationships (path *b*) are presented in Fig. 3. ACRD 5% increments were associated with a significant improvement in the rate (hazard) of all survival outcomes: 6% for OS (HR 0.94; 95% CI 0.91, 0.96; Fig. 3b), 4% for DFS (HR 0.96; 95% CI 0.92, 1.00; appendix SI 9), and 6% for CSS (HR 0.94; 95% CI 0.92, 0.96; appendix SI 9).

Conversely, ARDI increments of 5% were significantly associated with a worse OS rate (HR 1.05; 95% CI 1.01, 1.09; Fig. 3b), and a tendency towards worse DFS rate (HR 1.03; 95% CI 0.99, 1.07; appendix SI 9) and CSS rate (HR 1.03; 95% CI 1.00, 1.06; appendix SI 10).

There was minimal non-linearity demonstrated for all survival outcomes suggesting linear models were appropriate [appendix, SI 16].

Though we found no BMI-toxicity relationship, we sought to understand the association between G3+ toxicity, adherence and survival. Grade 3+ toxicity was associated with a significant reduction in both adherence measures (path *e*, Fig. 4): −10.37% for ACRD (95% CI −11.77, −8.97) and −3.86% for ARDI (95% CI −6.27, −1.45). Furthermore, G3+ toxicity was significantly associated with adverse OS (HR 1.37, 95% CI 1.17, 1.61; path *f*, Fig. 5) and DFS (HR 1.19, 95% CI 1.00, 1.43; path *f*, SI 11), and a tendency towards adverse CSS (HR 1.15; 95% CI 0.96;1.38; SI 12). Adjusting for ACRD (path *f* direct effect, Fig. 5) resulted in an approximate halving of effect estimates for OS and DFS, suggesting partial mediation via reduced ACRD (OS HR 1.20, 95% CI 1.02, 1.41; DFS HR 1.08, 95% CI 0.94, 1.22 [SI 11]). Effect estimates for CSS, almost completely attenuated (HR 1.02, 95% CI 0.85, 1.24 [SI 11]), suggesting near complete mediation. Conversely, adjusting for ARDI resulted in minimal change to effect estimates, indicating toxicity did not convincingly influence survival via ARDI.

Finally, assuming a linear relationship, we assessed the total effect of BMI on survival and found no evidence of an association (path *c*, Fig. 6): OS HR 0.98 (95% CI 0.90, 1.07), DFS HR 0.96 (95% CI 0.82, 1.12; appendix SI 13), or CSS HR 0.95 (95% CI 0.85, 1.06; appendix SI 14). Adjustment for ACRD (path *c* direct effect) appeared to reduce the HR by 11% for OS (HR 0.87, 95% CI 0.66, 1.13), though not statistically significant, this would suggest an indirect effect via reduced ACRD (given the significant BMI-ACRD and ACRD-OS relationships). However, individual trial HRs only reduced by approximately 1–4%, not explaining such a large change in effect. Trial weighting differed between total effect and ACRD-adjusted direct effect models. Hence, the larger-than-expected HR reduction was likely attributed to increased MOSAIC and PROCTORSCRIPT weighting. Repeating the direct effect analysis with weights fixed to those of the total effect model yielded a more plausible HR reduction of approximately 2% (HR 0.96 (95% CI 0.72, 1.29), appendix SI 15). Adjustment for ARDI resulted in minimal to no change in effect estimates, suggesting no indirect effect via ARDI.

There was minimal non-linearity seen for all survival outcomes suggesting linear models were appropriate [appendix, SI 16].

Meta-analysis of within-trial interaction terms demonstrated evidence of significant effect modification of path *a* BMI-ARDI relationships by sex (Coef._(interaction)_ 1.63; 95% CI 1.05, 2.20, appendix, SI 17), meaning women received smaller reductions in ARDI per 5 kg/m^2^ BMI increment. No effect modification by sex was demonstrated for BMI effect on ACRD, nor for any other path. Furthermore, there was no significant within-trial disease-stage interaction (appendix, SI 18).

Tau^2^ values demonstrated that heterogeneity tended to be low to moderate for most path analyses. Heterogeneity was higher for path *a* ACRD models, and particularly for the path e ARDI model.

Sensitivity analyses (appendix, SI 19–24) demonstrated mostly consistent relationships, with a few exceptions. For path *a*, the complete case analysis demonstrated the risk of bias and loss of statistical power of such analyses, with BMI-ACRD relationships not reaching significance (appendix, SI 19). Furthermore, exclusion of patients receiving capecitabine resulted in a larger effect estimate (Coef. −1.40; 95% CI −3.09, 0.30) with wider confidence intervals, and the conservative 95% confidence intervals produced by the Hartung-Knap-Siddik-Jonkmann (HKSJ) method widened the 95%CIs such that they just crossed the null effect (Coef. −1.15; 95% CI −2.41, 0.11).

Importantly, excluding early deaths resulted in a minor reduction of the strength of ACRD-OS (by approximately 1%, appendix SI 21), suggesting minimal reverse causality (i.e., early deaths during ACT resulting in reduced adherence did not substantially affect results). However, exclusion of early deaths approximately halved summary effect estimates for G3+ toxicity-OS relationships (HR 1.19; 95% CI 1.01, 1.40), as might be expected given that some of these deaths were directly attributed to ACT-related toxicity.

## Discussion

Our IPD meta-analysis found that 5 kg/m^2^ BMI increments were associated with substantial odds of being dose capped, equating to a 2% reduction in cycle 1 ARDR, and a modest but significant 1% reduction in ACRD and ARDI. This attenuation (2% cycle one dosing reduction to 1% overall adherence reduction) was due to longitudinal dose reductions, decreasing initial differences across BMI (data not shown). Non-linear associations demonstrated that these relationships begin at BMI > 25Kg/m^2^, and that linear parameter estimates might be underestimated (i.e., true effect estimates may be larger than those reported). Two studies have previously reported a proportional BMI-dose-capping relationship in adjuvant [9] and metastatic [10] CRC settings. However, two additional studies in colon [21] and rectal [22] cancer reported low overall dose capping incidence unrelated to BMI. Data on BMI-adherence relationships is lacking within the CRC literature, with little “real-world” data on dose capping prevalence outside of clinical trials.

We found no evidence to support dose-capping on safety grounds, with no significant BMI-G3+ toxicity relationship, and furthermore, no effect modification of this relationship by dose-capping. Hence, toxicity was unlikely to mediate BMI-adherence and BMI-survival relationships. Our findings are comparable with previous studies demonstrating reduced G3+ toxicity or no significant relationship in patients receiving fully dosed chemotherapy for CRC in adjuvant [21, 22] and metastatic settings [10, 34].

The prognostic benefits of maintaining a high dose, dose intensity or dose density have mainly been demonstrated within the breast cancer literature [35, 36]. CRC is comparatively less chemo-sensitive, and dose modifications/delays have been previously reported to not adversely affect survival within an ACT trial [37]. However, within the CRC obesity literature, prognostic data on adherence are lacking. Two studies of adjuvant [11] and metastatic [10] CRC demonstrated that reduced dosing in obese patients was associated with poorer outcomes, but are limited by exclusion of non-obese patients. Conversely, dose-capping as a binary indicator has tended not to display any significant relationship with survival, though may not be sufficiently discriminative as a binary variable [9, 21, 22]. Our study reports the novel observation of the relative importance of optimising ACRD compared with ARDI. We found a significant 6% improvement in OS with 5% increments of ACRD, contrary to the inversely proportional and less convincing relationship between ARDI and OS. Whilst it is possible that dose intensity is less important than the cumulative dose, these differences more likely represent discriminative limitations of ARDI in the context of early treatment discontinuation (as discussed in the methods).

The significant association between G3+ toxicity and reduced adherence was largely anticipated, given protocol-mandated toxicity-related dose reductions. We found novel evidence that G3+ toxicity might adversely affect OS both directly and indirectly via reduced ACRD. Grade 3+ toxicity was associated with an increased risk of death for OS, with results attenuating on adjusting for ACRD, suggesting almost half of this effect might be mediated through ACRD reductions. Conversely to our findings, some data exist supporting the alternative concept of toxicity as a surrogate of optimal dosing and improved survival for capecitabine or 5FU in metastatic [38, 39] and non-metastatic CRC [40, 41]. These studies were limited by lack of dosing data, and small numbers of high-grade toxicities [38–41] and therefore such observations may be more applicable to lower grade toxicity.

Three large studies have demonstrated a modest increased risk of adverse survival outcomes with increasing BMI, particularly BMI ≥ 35 kg/m [2, 9, 42, 43]. A further three aggregate data meta-analyses have examined BMI-CRC relationships [3, 4, 44], demonstrating U-shaped BMI and overall survival/mortality relationships. The recent AICR Global Cancer Update Project reported a reverse J-shaped relationship for BMI with the highest risk of all-cause mortality, colorectal cancer specific mortality, and cancer recurrence/DFS at both ends of the BMI distribution (18 and 38 kg/m^2^) [7]. An additional systematic review and meta-analysis from our group, previously attempted to deal with some of the issues of heterogeneity by grouping analyses by study type and reported insufficient evidence for consistent BMI-mortality/survival relationships [2]. Finally, a post-hoc analysis of the TOSCA trial reported a significant association between BMI > 30 vs. <25 kg/m^2^ and adverse OS (HR 1.57, 95% CI 1.00, 2.47) in univariable analysis for 1455 patients (of the total 3759 study population) [45]. However, all of these studies were limited by the inability to account for and characterise variations in chemotherapy dosing and toxicity effects. In addition, may studies either had high levels of missing data or did not report on missing data, dichotomised or categorised BMI (resulting in loss of information), did not account for heterogeneity, confounding and selection bias, and are likely to have been substantially biased by reverse causality.

Despite a possible adverse indirect effect, we found no significant BMI-survival relationship. Adjusting for ACRD resulted in a large change in the effect estimate, however, this likely resulted from the change in trial-level weights between models. Hence, the possible small indirect effect did not appear to be large enough to adversely influence the total effect. Despite BMI-incident CRC risks, reassuringly, it seems that CRC survival is not influenced by elevated BMI, at least in the setting of ACT-RCTs.

Given sex differences in obesity-related CRC incidence and stage-related differences reported by SCOT, we explored effect modification by sex and stage. We found no significant within-trial BMI-sex interaction for ACRD relationships, and no effect modification by stage.

There are several strengths to our study. First, RCT data were high quality, with reduced bias from protocol-directed treatment, follow-up, and data collection. Second, clinical and pathological characteristics and cycle-level dosing and toxicity data were available, facilitating detailed analysis and adequate confounder adjustment. Third, the causal inference approach facilitated confounder selection, presentation of assumptions and exploration of potential bias, including eliminating toxicity as an intermediate confounder (no BMI-toxicity relationship). Fourth, multiple imputation reduced bias from missing toxicity data. Fifth, we avoided categorising continuous exposures, preventing information loss and assessed non-linearities. Finally, the IPD meta-analysis approach inherently strengthened our study through data harmonisation, standardisation of analyses, a large sample to enable detection of the impact of relatively small reductions in adherence on medium-term outcomes [46], reducing the risk of type II error, whilst accounting for within and across trial heterogeneity.

We also encountered a number of limitations. BMI is an imperfect measure of body composition and may not adequately represent life-time exposure at trial entry, risking measurement error, exposure misclassification and reverse causality [47]. Though the latter was explored in sensitivity analyses, other anthropometric measures and pre-diagnosis BMI were not available [43, 44]. Residual confounding may exist, e.g., due to lack of available smoking data. Furthermore, though we attempted to address the large volume of missing toxicity data from the SCOT trial through utilisation of MI methods, with focus on approaches to evaluate and reduce the risk of bias (e.g., sensitivity analyses and assessment of missingness), there may have been residual confounding or bias within effect estimates. Summarising toxicity data as a grade 3+ indicator variable, though necessary for data-harmonisation reasons (as CHRONICLE only provided grade 3+ toxicity data), may not have captured the extent of dosing-toxicity-adherence relationships, particularly where cumulative low-grade toxicity may have resulted in dose alterations. However, important toxicities such as neuropathy and neutropenia were reported by the individual trials and included within the composite variable. Additionally, we were not able to account for oral anti-neoplastic adherence, and recognise the possibility of both under-and over- compliance. Whether data from RCTs fully represent wider oncology populations due to selection into trials [48], and thus the possibility of a “healthy obese” bias remains. However, a substantial proportion of data came from SCOT, and though regimen selection was non-randomised, it was a pragmatic trial, with a large range in BMI and several contributing sites using modern dosing regimens, and was likely to be representative of modern-day dosing [17]. For analyses additionally adjusting for a mediator, there remains a risk of introducing collider stratification bias [49, 50]. Finally, we were not able to explore biological characteristics such as tumour sidedness, BRAF, KRAS, NRAS or DNA miss match repair/microsatellite instability status, as data were not available.

The updated American Society of Clinical Oncology guidance [46] on systemic therapy dosing for obese adult patients with cancer recommends full BSA-based dosing of cytotoxic chemotherapy irrespective of obesity status. However, this was based on low quality evidence. Our findings provide the highest level of evidence to date in support of full BSA-based dosing, within the context of non-metastatic CRC, both on the grounds of efficacy and safety, and contribute substantially to understanding how relationships might occur.

Though effect estimates for BMI-adherence and adherence-survival relationships were small, the higher the BMI, the more clinically relevant these effects become. In practice, however, dosing decisions evaluate an individual patient’s risk-benefit profile, where well-established factors may necessitate dose reductions for safety (e.g., reduced renal function or dihydropyrimidine dehydrogenase (DPD) deficiency).

Our study is pertinent to planning future studies/trials in the wider oncology setting, stressing the importance of understanding treatment effects for obese patients with cancer. Efforts to ensure adequate representation, accurate measurement, and planned reporting of secondary adiposity-related outcomes should be considered routinely in clinical studies, given the increasing rates of obesity. Additionally highlighted are the potential issues concerning reliance on ARDI, particularly where there is early drug discontinuation, and we encourage investigators to consider whether ACRD could better contextualise findings. Finally, we demonstrate the utility of the causal inference approach in clinical oncology for understanding putative causal paths and indirect treatment effects.

Clinical trial data may not be applicable to wider oncology populations [51], hence, population-based studies may be required to contextualise our findings. Further work is required to explore how body composition (particularly sarcopenic obesity) and pharmacokinetics relate to the relationships presented. Formal mediation analysis can quantify indirect, direct and total effects, and though meta-mediation approaches are emerging, they require further exploration and validation.

In conclusion, our study has established the possibility of a modest adverse indirect effect of BMI on survival, acting through treatment-selection (dose capping and reduction in ACT adherence), supporting the concept of full weight-based ACT dosing in CRC. We have defined putative causal paths, providing an additional layer of understanding to complex BMI-chemotherapy-toxicity-survival relationships, which may support decision-making and informed discussions with patients.

## Supplementary information

Supplementary Items

PRISMA IPD Checklist

## Data Availability

Data used for this study are not owned by the authors. MOSAIC (control arm only) was publicly available from Project Datasphere (https://data.projectdatasphere.org/projectdatasphere/html/home). Data from SCOT, CHRONICLE and PROCTORSCRIPT were made available to the authors through data transfers, and as such are not publicly published.
