# Supplementary material for: Body mass index, adjuvant chemotherapy, toxicity, and survival in non-metastatic colorectal cancer: an individual participant data meta-analysis (OCTOPUS)
Source: Br J Cancer. 2026 Jun 9;135(5):773–83. doi: 10.1038/s41416-026-03472-4 (PMC13478639; doi:10.1038/s41416-026-03472-4)
Supplement: Supplementary file 2 [file 41416_2026_3472_MOESM1_ESM.docx]

**Supplementary items: Table of contents**

[SI1 | Summary of risk of bias assessment and data quality	2](#_Toc137743791)

[SI2 | Flow Chart	3](#_Toc137743792)

[SI3 | Table summarising trial chemotherapy regimens	4](#_Toc137743793)

[SI4 | Definitions and calculations of chemotherapy-related adherence measures	5](#_Toc137743794)

[SI5 | Directed Acyclic Graph (DAG) demonstrating the hypothesised causal pathways.	6](#_Toc137743795)

[SI6 | Trial Characteristics	7](#_Toc137743796)

[SI7 | Effect of 5kg/m^2^ increments of BMI on dose capping and ARDR	8](#_Toc137743797)

[SI8 | Effect of BMI-dose capping interactions on grade 3+ toxicity occurrence.	9](#_Toc137743798)

[SI9 | Path *b* – Effect of 5% increments of ACRD and ARDI on disease free survival (DFS)	10](#_Toc137743799)

[SI10 | Path *b*– Effect of 5% increments of ACRD and ARDI on cancer specific survival (CSS)	11](#_Toc137743800)

[SI11 | Path f – Total and direct (adjusting for ACRD and ARDI) effects of grade 3+ toxicity on disease free survival (DFS)	12](#_Toc137743801)

[SI12 | Path f - Total and direct (adjusting for ACRD and ARDI) effects of grade 3+ toxicity on cancer specific survival (CSS)	13](#_Toc137743802)

[SI13 | Path C – Total and direct (adjusting for ACRD and ARDI) effects of 5kg/m^2^ BMI increments on disease free survival (DFS)	14](#_Toc137743803)

[SI14 | Path C – Total and direct (adjusting for ACRD and ARDI) effects of 5kg/m^2^ BMI increments on cancer specific survival (CSS)	15](#_Toc137743804)

[SI15 | Path *c* with fixed weights – Total and direct (adjusting for ACRD and ARDI) effects of 5kg/m^2^ BMI increments on overall survival (OS)	16](#_Toc137743805)

[SI16 | Non-linear models	17](#_Toc137743806)

[SI17 | Exposure-sex interaction models.	18](#_Toc137743807)

[SI18 | Exposure-stage interaction models.	19](#_Toc137743808)

[SI19 | Path *a* total effect sensitivity analysis	20](#_Toc137743809)

[SI20 | Path *d* total effect sensitivity analysis	21](#_Toc137743810)

[SI21 | Path *b* total effect sensitivity analysis (overall survival)	22](#_Toc137743811)

[SI22 | Path *e* total effect sensitivity analysis	23](#_Toc137743812)

[SI23 | Path *f* total and direct effects sensitivity analysis (overall survival)	24](#_Toc137743813)

[SI24 | Path *c* total and direct effects sensitivity analysis (overall survival)	25](#_Toc137743814)

| SI1 \| Summary of risk of bias assessment and data quality  Summary risk of bias judgements following use of the Risk of Bias - Version 2 (RoB2) assessment tool for meta-analysis of randomised trials from Sterne et al.^17^ Due to the nature of this study, not all domains were applicable. Furthermore, assessment of certain domains was extended to include evaluation of exposure, confounders, and mediators in addition to outcomes, in addition to including judgement on data quality. Of note, there was a moderate risk of bias due to missing data, pertaining mainly to the SCOT trial and the protocolised collection of toxicity data for only the first 848 patients. Hence, multiple imputation approaches were adopted to limit potential biases resulting from complete case analysis. | |
| --- | --- |
| **Bias arising from the randomisation process** | |
| **Low risk** | *Randomisation not directly applicable to analyses as exposure cannot be randomised. All trials followed randomised allocation (open label), with no evidence of baseline imbalance. Therefore, randomisation was likely to reduce bias within study context, even though studied exposure cannot be randomised.* |
| **Bias due to deviations from intended interventions** | |
|  | *Section not applicable as study specifically assesses deviation variability in dosing related to BMI, and these effects on outcomes.* |
| **Bias due to missing outcome data (extended to include exposure, confounders, and mediators)** | |
| **Low risk**  **–**  **Exposure** | *Proportion of eligible patients with missing BMI data was low, and unlikely to substantially influence outcomes. BMI data quality was good overall.* |
| **Low/moderate risk**  **-**  **Confounders** | *Minimal missing data for key confounders. Additional confounders such as poor prognostic features (e.g., lymph node harvest), race, smoking status mostly systematically missing and could not be accounted for.* |
| **Low risk**  **–**  **Adherence measures** | *Minimal missing chemotherapy data (11 patients excluded overall due to non-calculable adherence measures (either missing cycle dose or date data).* |
| **Moderate risk**  **–**  **Toxicity** | *Missingness relates mainly to toxicity data – potential bias resulting from missing toxicity dat in SCOT; however, this was protocol-related and therefore unlikely to be due to other study factors.* |
| **Low risk**  **–**  **Survival** | *There was evidence that survival outcomes were not biased by missing toxicity data.* |
| **Bias in measurement of the outcome (extended to include exposure, confounders, and mediators)** | |
| **Low risk**  **–**  **Exposure** | *Quality of BMI data appeared good overall, height and weight assumed to be directly measured and recorded as part of. Minor apparent data-entry errors identified, and sensitivity analysis planned to evaluate potential effects.* |
| **Low risk**  **-**  **Confounders** | *No significant issues identified of pertaining to measurement of confounders.* |
| **Low risk**  **–**  **Adherence measures** | *Quality of chemotherapy data overall good, minor proportion of patients with apparent data-entry errors and sensitivity analysis planned to evaluate potential effects.* |
| **Low/moderate risk**  **–**  **Toxicity** | *Trials graded toxicity according to standardised Common Terminology Criteria of Adverse Events (though unclear whether this was used for PROCTOR-SCRIPT).* |
| **Low risk**  **–**  **Survival** | *No concerns identified regarding survival outcome measurement. Issues regarding sufficient follow-up duration/attrition identified within all trials to a degree. Bias risk reduced by time-to-event modelling. Outcomes applicable for median follow-up of 3 years.* |
| **Bias in selection of the reported result** | |
|  | *Not directly applicable as not an aggregate data meta-analysis.*  *All a priori defined analyses are presented, including null results.*  *Data for all a priori defined outcomes where available.* |
| **Overall bias** | |
| **Low risk** | *Overall, good quality of datasets, some concerns over missing toxicity data addressed through multiple imputation methods and sensitivity analyses.* |

SI2 | Flow Chart

*Control arm only available from project DataSphere

† 55 patients withdrew from SCOT, therefore no randomisation arm or chemotherapy data

§ TOX1 population excludes any patients who did not have protocol-mandated toxicity data collected i.e. were in the trial “safety” populations (exclusions apply to the SCOT trial only)

‡ TOX2 population included patients with available toxicity data this included an additional 921 patients who had ‘ad hoc’ toxicity data collected despite not being in the SCOT trial “safety” population

| SI3 \| Table summarising trial chemotherapy regimens | | | | | | | | | | | |
| --- | --- | --- | --- | --- | --- | --- | --- | --- | --- | --- | --- |
| **Trial** | **Regimen** | **Drug** | **Dose/m^2^** | **Dosing/Duration** | **Expected cycle dose/m^2^** | **No. Cycles** | **Dosing interval** | **Expected cumulative dose/m^2^** | **Expected duration (weeks)** | **Expected**  **Dose intensity**  **(mg/ m^2^/week)** |  |
| **MOSAIC** | 5FU/LV2 | 5FU-B | 400mg | Day 1 (bolus)  Day 2 (bolus) | 2000mg | 12 | 14 days | 24,000mg | 24 | 1000 |  |
|  |  | 5FU-I | 600mg | Day 1 (22hrs)  Day 2 (22hrs) |  | 12 |  |  |  |  |  |
| **SCOT** | mFOLFOX6 | 5FU-B | 400mg | Day 1 (bolus) | 2800mg |  | 14 days | 16,800mg 33,600mg* |  |  |  |
|  |  | 5FU-I | 2400mg | Day 1 – 2 (46hrs) |  | 6 or 12* |  |  | 12 or 24* | 1400 |  |
|  |  | OX | 85mg | Day 1 (2hrs) | 85mg |  | 14 days | 510mg or 1020mg* |  | 42.5 |  |
|  | CAPOX | CAP | 1000mg | Days 1-14 (BD) | 28,000mg | 4 or 8* | 21 days | 112,000 or 224,000 | 12 or 24* | 9,333 |  |
|  |  | OX | 135mg | Day 1 (2hrs) | 135mg |  | 21 days | 540mg or 1,080mg |  | 45 |  |
| **CHRONICLE** | CAPOX | CAP | 1000mg | Days 1-14 (BD) | 28,000mg | 6 | 21 days | 168,000mg | 18 | 9,333 |  |
|  |  | OX | 135mg | Day 1 (2hrs) | 135mg | 6 | 21 days | 810mg |  | 45 |  |
| **PROCTOR** | MAYO | 5FU | 435mg | Days 1-5 (bolus) | 2,175mg | 6 | 4-5 weeks | 13,050mg | 25.7^†^ | 507.5 |  |
|  | NORDIC | 5FU: | 500mg | Days 1-2 (bolus) | 1,000mg | 12 | 14 days | 12,000mg | 24 | 500 |  |
| **SCRIPT** | CAP | CAP | 1250mg | Days 1-14 (BD) | 35,000mg | 8 | 21 days | 280,000mg | 24 | 11,667 |  |
| Abbreviations: 5FU, 5 Fluorouracil; 5FU-B, 5FU bolus; BD, twice daily; CAP, Capecitabine; I, 5FU infusion; OX, oxaliplatin;  *3 months vs. 6 months arms arm.  ^†^ Dosing interval of 30 days assumed (4-5 weeks) for 6 cycles to calculate total expected duration | | | | | | | | | | | |

| SI4 \| Definitions and calculations of chemotherapy-related adherence measures | | |
| --- | --- | --- |
| **Measure** | **Units** | **Definition** |
| Actual cycle dose (ACD) received | mg/m^2^ | Total cycle dose (mg) / BSA (m^s^) |
| Expected cycle dose (ECD) | mg/m^2^ | Cycle dose according to trial protocol in mg/m^2^ (see tableS2) |
| Relative cycle dose received (RDR) | % | (ACD [mg/m^2^] / ECD [mg/m^2^]) x 100 |
| Average relative cycle dose received (ARDR) | % | Sum of RDR for all drugs in the regimen / number of drugs in regimen |
| Dose capping | - | Receipt of <95% RDR for cycle 1 |
| Actual number of cycles | - | Number of cycles of chemotherapy where at least one cytotoxic drug was administered |
| Expected number of cycles | - | Number of cycles of expected to be delivered according to the trial protocol (see table S2) |
| Actual cumulative dose | mg/m^2^ | Sum of ACD for all cycles received for any given drug |
| Expected cumulative dose | mg/m^2^ | ECD x expected number of cycles for the regimen |
| Cumulative relative dose (CRD) | % | (Actual cumulative dose / expected cumulative dose [mg/m^2^]) x 100 |
| Average cumulative relative dose (ACRD) | % | Sum of CRD for all drugs in the regimen / number of drugs in the regimen |
| Actual dose intensity | mg/m^2^/week | Actual cumulative dose (mg/m^2^) / actual treatment duration (weeks) |
| Expected dose intensity | mg/m^2^/week | Expected cumulative dose (mg/m^2^) / expected treatment duration (weeks) |
| Relative dose intensity (RDI) | % | (Actual dose intensity / expected dose intensity) x 100 |
| Average relative dose intensity (ARDI) | % | Sum of RDI for all drugs in the regimen / number of drugs in the regimen |

SI5 | Directed Acyclic Graph (DAG) demonstrating the hypothesised causal pathways.

The main mediated pathway is demonstrated in **blue**: BMI (**exposure, X**) is associated with survival (**outcome, Y**) both directly (**path c**) and indirectly (**path ab**) via chemotherapy adherence (**mediator, M**). Baseline confounders (**yellow**) must be a cause of both exposure and outcome e.g. worse disease stage may cause both weight loss and worse survival. Confounders open up backdoor pathways between exposure and outcome (e.g. BMI-Age-survival), potentially introducing spurious relationships, and adjusting for them blocks these pathways. Here we assume that all baseline confounders affect all pathways, and hence the same confounders are included in all analyses. Intermediate confounders (**L, purple**) are a mediator of both X-M (**path a**) and X-Y (**path c**) relationships, and a confounder of M-Y (**path b**) relationships, and hence complicate mediated pathways. Unmeasured confounding (**U, red arrows**), may produce biased estimates, and limit the interpretation of causality due to residual confounding, in addition to the potential to open up backdoor pathways as a result of collider effects, where unmeasured confounding involves mediated pathways. Note that when looking at the pathways independently, the role of the variables may change. For example when the interested in only the relationship between chemotherapy adherence and survival (path b), both BMI and toxicity are confounders (causes of) this relationship.

| SI6 \| Trial Characteristics | | | | | | | | | | | | | |
| --- | --- | --- | --- | --- | --- | --- | --- | --- | --- | --- | --- | --- | --- |
|  | **Colorectal Cancer Trials** | | | | | | | | | | | | |
|  | **MOSAIC** | | **SCOT_3M** | | **SCOT_6M** | | **CHRONICLE** | | **PS** | | | **Total** | |
|  | **N=1,097** | | **N=2,965** | | **N=2,978** | | **N=49** | | **N=175** | | | **N=7,264** | |
| **Recruitment period** | 1998 - 2001 | | 2008-2013 | | 2008-2013 | | 2004-2008 | | 2000-2013 | | NA | | |
| **BMI, median kg/m^2^ (IQR)** | 24.68 | (22.34-27.72) | 26.24 | (23.63-29.39) | 26.51 | (23.57-29.68) | 24.73 | (23.03-27.60) | 24.57 | (22.28-26.79) | 26.01 | | (23.31-29.22) |
| **BMI category** |  |  |  |  |  |  |  |  |  |  |  | |  |
| **Underweight** | 36 | (3.3%) | 42 | (1.4%) | 36 | (1.2%) | 0 | (0.0%) | 1 | (0.6%) | 115 | | (1.6%) |
| **Normal** | 542 | (49.4%) | 1,069 | (36.1%) | 1,071 | (36.0%) | 27 | (55.1%) | 98 | (54.9%) | 2,807 | | (38.6%) |
| **Overweight** | 384 | (35.0%) | 1,211 | (40.8%) | 1,191 | (40.0%) | 17 | (34.7%) | 72 | (40.0%) | 2,875 | | (39.6%) |
| **Obese** | 135 | (12.3%) | 643 | (21.7%) | 680 | (22.8%) | 5 | (10.2%) | 9 | (5.0%) | 1,472 | | (20.3%) |
| **BSA, median m^2^(IQR)** | 1.79 | (1.65-1.93) | 1.90 | (1.75-2.06) | 1.90 | (1.74-2.06) | 1.79 | (1.71-2.02) | 1.89 | (1.73-2.01) | 1.88 | | (1.72-2.04) |
| **Age, median years (IQR)** | 60.00 | (52.00-67.00) | 65.00 | (58.00-70.00) | 65.00 | (58.00-70.00) | 60.00 | (55.00-67.00) | 60.99 | (55.67-67.34) | 64.00 | | (57.00-69.00) |
| **Sex, N (%)** |  |  |  |  |  |  |  |  |  |  |  | |  |
| **Male** | 575 | (52.4%) | 1,793 | (60.5%) | 1,807 | (60.7%) | 40 | (81.6%) | 109 | (60.6%) | 4,324 | | (59.5%) |
| **Female** | 522 | (47.6%) | 1,172 | (39.5%) | 1,171 | (39.3%) | 9 | (18.4%) | 71 | (39.4%) | 2,945 | | (40.5%) |
| **WHO performance status, N (%)** |  |  |  |  |  |  |  |  |  |  |  | |  |
| **0** | 335 | (30.5%) | 2,134 | (72.0%) | 2,098 | (70.4%) | 30 | (61.2%) | 116 | (64.4%) | 4,713 | | (64.8%) |
| **1** | 627 | (57.2%) | 831 | (28.0%) | 880 | (29.6%) | 19 | (38.8%) | 59 | (32.8%) | 2,416 | | (33.2%) |
| **2+** | 135 | (12.3%) | 0 | (0.0%) | 0 | (0.0%) | 0 | (0.0%) | 5 | (2.8%) | 140 | | (1.9%) |
| **Race, N (%)** |  |  |  |  |  |  |  |  |  |  |  | |  |
| **White** | 1,077 | (98.2%) | 2,427 | (81.9%) | 2,482 | (83.3%) |  | NR |  | NR | 5,986 | | (82.3%) |
| **Other** | 20 | (1.8%) | 109 | (3.7%) | 76 | (2.6%) |  | NR |  | NR | 205 | | (2.8%) |
| **Missing** | 0 | (0.0%) | 429 | (14.5%) | 420 | (14.1%) | 49 | (100.0%)* | 180 | (100.0%)* | 1,078 | | (14.8%) |
| **Disease site, N (%)** |  |  |  |  |  |  |  |  |  |  |  | |  |
| **Colon** | 1,097 | (100.0%) | 2,430 | (82.0%) | 2,439 | (81.9%) | 0 | (0.0%) | 0 | (0.0%) | 5,966 | | (82.1%) |
| **Rectum** | 0 | (0.0%) | 535 | (18.0%) | 539 | (18.1%) | 49 | (100.0%) | 180 | (100.0%) | 1,303 | | (17.9%) |
| **Tumour-stage, N (%)** |  |  |  |  |  |  |  |  |  |  |  | |  |
| **(y)pT0-2** | 61 | (5.6%) | 370 | (12.5%) | 370 | (12.4%) | 23 | (46.9%) | 40 | (22.2%) | 864 | | (11.9%) |
| **(y)pT3** | 832 | (75.8%) | 1,698 | (57.3%) | 1,709 | (57.4%) | 24 | (49.0%) | 138 | (76.7%) | 4,401 | | (60.5%) |
| **(y)pT4** | 204 | (18.6%) | 897 | (30.3%) | 899 | (30.2%) | 2 | (4.1%) | 2 | (1.1%) | 2,004 | | (27.6%) |
| **Nodal-stage, N (%)** |  |  |  |  |  |  |  |  |  |  |  | |  |
| **(y)pN0** | 443 | (40.4%) | 546 | (18.4%) | 541 | (18.2%) | 40 | (81.6%) | 39 | (21.7%) | 1,609 | | (22.1%) |
| **(y)pN1** | 433 | (39.5%) | 1,686 | (56.9%) | 1,692 | (56.8%) | 6 | (12.2%) | 90 | (50.0%) | 3,907 | | (53.7%) |
| **(y)pN2** | 221 | (20.1%) | 733 | (24.7%) | 745 | (25.0%) | 3 | (6.1%) | 51 | (28.3%) | 1,753 | | (24.1%) |
| **Dose Capped, N (%)** |  |  |  |  |  |  |  |  |  |  |  | |  |
| **Underweight**  **Normal**  **Overweight**  **Obese**  **Total** | 0  12  17  40  69 | (0,0%)  (2.2%)  (4.4%)  (29.6%)  (6.3%) | 9  284  400  400  1093 | (21.4%)  (26.6%)  (33.0%)  (62.2%)  (36.85%) | 8  297  434  418  1157 | (22.2%)  (27.7%)  (36.4%)  (61.5%)  (38.85%) | -  8  10  3  21 | -  (29.6%)  (58.8%)  (60.0%)  (38.85) | 0  24  23  5  52 | (0.0%)  (25.0)  (33.3%)  (55.56)  (29.71%) | 17  625  884  866  2392 | | (14.8%)  (22.3%)  (30.8%)  (58.8%)  (32.9%) |
| **Grade 3+ Toxicity, N (%)** |  |  |  |  |  |  |  |  |  |  |  | |  |
| **No**  **Yes**  **Missing** | 758  339  0 | (69.10%)  (30.9%)  (0.0%) | 595  290  2080 | ( 20.07%)  (9.78%)  (70.2%) | 471  415  2092 | (15.82%)  (13.94%)  (70.2%) | 30  19  0 | (61.22%)  (38.78%)  (0.0%) | 104  71  0 | (26.9%)  (40.57%)  (0.0%) | 1958  1134  4172 | | (26.95%)  (15.61%)  (57.43%) |
| **Abbreviations: BMI,** Body Mass Index; **BSA**, Body Surface Area; **NR**, not recorded; **WHO**, World Health Organisation  *Not recorded (systematically missing) | | | | | | | | | | | | | |

SI7 | Effect of 5kg/m^2^ increments of BMI on dose capping and ARDR

SI8 | Effect of BMI-dose capping interactions on grade 3+ toxicity occurrence.

a) Overall effect of 5kg/m^2^ increments of BMI with interaction term fitted in model b) Within trial interaction effect.

SI9 | Path *b* – Effect of 5% increments of ACRD and ARDI on disease free survival (DFS)

SI10 | Path *b*– Effect of 5% increments of ACRD and ARDI on cancer specific survival (CSS)

SI11 | Path f – Total and direct (adjusting for ACRD and ARDI) effects of grade 3+ toxicity on disease free survival (DFS)

SI12 | Path f - Total and direct (adjusting for ACRD and ARDI) effects of grade 3+ toxicity on cancer specific survival (CSS)

SI13 | Path C – Total and direct (adjusting for ACRD and ARDI) effects of 5kg/m^2^ BMI increments on disease free survival (DFS)

SI14 | Path C – Total and direct (adjusting for ACRD and ARDI) effects of 5kg/m^2^ BMI increments on cancer specific survival (CSS)

SI15 | Path *c* with fixed weights – Total and direct (adjusting for ACRD and ARDI) effects of 5kg/m^2^ BMI increments on overall survival (OS)

SI16 | Non-linear models

Effect of BMI on **a)** dose capping; **b)** ARDR; **c)** ARDI (path *a)*; **d)** ACRD (path *a)*; **e)** grade 3+ toxicity (path *e)*. Effect of ARDI on **f)** overall survival (path *b)*; **g)** disease-free survival (path *b)*; **h)** cancer specific survival (path *b)*; Effect of ACRD on **i)** overall survival (path *b)*; **j)** disease-free survival (path *b)*; **k)** cancer specific survival (path *b)***;** Effect of BMI on **l)** overall survival (path *c)*; **m)** disease-free survival (path *c)*; **n)** cancer specific survival (path *c)*

**a)	b)**

**c)	d)	e)**

**f)	g)	h)**

**i)	j)	k)**

**l)	m)	n)**

| SI17 \| Exposure-sex interaction models.  Meta-analysed summary effects for the effect of exposure (X) on outcome (Y) and the interaction terms, when including exposure-sex interaction within the total effect path models. Effect estimates presented as *Odds ratio; ^†^Coef; or ^‡^Hazard ratios depending on the path | | | | | | | | | | |  |
| --- | --- | --- | --- | --- | --- | --- | --- | --- | --- | --- | --- |
|  |  |  | **Exposure effect** | | | | **Interaction effect** | | | | |
|  |  |  |  |  |  |  | **(Female vs. Male)** | | | | |
| **Path** | **Y** | **X** | **Est*^†‡^** | **(95%CI)** | | **Tau2** | **Est*^†‡^** | **(95%CI)** | | **Tau2** | |
| ***NA*** | **Capping*** | **BMI** | **3.01** | **(2.07,** | **4.38)** | 0.030 | **0.60** | **(0.53,** | **0.68)** | 0.000 | |
|  | **ARDR^†^** | **BMI** | **-2.63** | **(-3.31,** | **-1.94)** | 0.488 | **1.17** | **(0.34,** | **2.01)** | 0.634 | |
| ***a*** | **ARDI^†^** | **BMI** | **-1.82** | **(-2.55,** | **-1.09)** | 0.283 | **1.54** | **(0.88,** | **2.19)** | 0.073 | |
|  | **ACRD^†^** | **BMI** | **-1.38** | **(-2.35,** | **-0.41)** | 0.282 | 0.82 | (-0.31, | 1.95) | 0.162 | |
| ***d*** | **G3 Tox*** | **BMI** | 0.98 | (0.88, | 1.09) | 0.000 | 1.04 | (0.91, | 1.19) | 0.000 | |
| ***b*** | **OS^‡^** | **ARDI** | 1.04 | (1.00, | 1.09) | 0.000 | 1.02 | (0.97, | 1.08) | 0.000 | |
|  | **DFS^‡^** | **ARDI** | 1.01 | (0.96, | 1.07) | 0.002 | 1.04 | (0.98, | 1.10) | 0.001 | |
|  | **CSS^‡^** | **ARDI** | 1.01 | (0.96, | 1.05) | 0.000 | 1.04 | (0.98, | 1.11) | 0.000 | |
|  | **OS^‡^** | **ACRD** | **0.93** | **(0.91,** | **0.96)** | 0.000 | 1.00 | (0.97, | 1.03) | 0.000 | |
|  | **DFS^‡^** | **ACRD** | **0.95** | **(0.93,** | **0.98)** | 0.000 | 0.99 | (0.97, | 1.01) | 0.000 | |
|  | **CSS^‡^** | **ACRD** | **0.95** | **(0.92,** | **0.98)** | 0.000 | 0.98 | (0.95, | 1.03) | 0.000 | |
| ***e*** | **ARDI** | **G3+ Tox** | **-3.53** | **(-5.41,** | **-1.64)** | 2.152 | 1.54 | (0.88, | 2.19) | 0.073 | |
|  | **ACRD** | **G3+ Tox** | **-10.59** | **(-12.64,** | **-8.54)** | 0.000 | 0.82 | (-0.31, | 1.95) | 0.162 | |
| ***f*** | **OS^‡^** | **G3+ Tox** | **1.42** | **(1.13,** | **1.77)** | 0.000 | 0.91 | (0.65, | 1.26) | 0.000 | |
|  | **DFS^‡^** | **G3+ Tox** | 1.19 | (0.93, | 1.52) | 0.030 | 0.98 | (0.77, | 1.26) | 0.000 | |
|  | **CSS^‡^** | **G3+ Tox** | 1.17 | (0.90, | 1.52) | 0.000 | 0.94 | (0.63, | 1.40) | 0.000 | |
| ***c*** | **OS^‡^** | **BMI** | 0.96 | (0.86, | 1.06) | 0.000 | 1.05 | (0.92, | 1.21) | 0.000 | |
|  | **DFS^‡^** | **BMI** | 0.99 | (0.85, | 1.15) | 0.015 | 0.98 | (0.88, | 1.09) | 0.000 | |
|  | **CSS^‡^** | **BMI** | 0.94 | (0.83, | 1.06) | 0.000 | 1.04 | (0.88, | 1.23) | 0.000 | |

| SI18 \| Exposure-stage interaction models.  Meta-analysed summary effects for the effect of exposure (X) on outcome (Y) and the interaction terms, when including exposure-stage interaction within the total effect path models. Effect estimates presented as *Odds ratio; ^†^Coef; or ^‡^Hazard ratios depending on the path | | | | | | | | | | | | | | | |
| --- | --- | --- | --- | --- | --- | --- | --- | --- | --- | --- | --- | --- | --- | --- | --- |
|  |  |  | **Exposure effect** | | | | **Interaction effect** | | | | **Interaction effect** | | | | |
|  |  |  |  |  |  |  | **Stage II-HR vs. Stage III-LR** | | | | **Stage III-HR vs. Stage III-LR** | | | | |
| **Path** | **Y** | **X** | **Est*^†‡^** | **(95%CI)** | | **Tau^2^** | **Est*^†‡^** | **(95%CI)** | | **Tau^2^** | **Est*^†‡^** | **(95%CI)** | | **Tau^2^** |  |
| ***NA*** | **Capping*** | **BMI** | 2.81 | (2.08, | 3.79) | 0.059 | 0.96 | (0.75, | 1.21) | 0.000 | 0.89 | (0.77, | 1.02) | 0.000 |  |
|  | **ARDR^†^** | **BMI** | -2.14 | (-2.59, | -1.69) | 0.131 | 0.08 | (-0.25, | 0.40) | 0.000 | 0.06 | (-0.19, | 0.32) | 0.000 |  |
| ***a*** | **ARDI^†^** | **BMI** | -1.35 | (-1.99 | -0.71) | 0.163 | 0.72 | (-0.17 | 1.60) | 0.000 | 0.28 | (-0.34 | 0.90) | 0.000 |  |
|  | **ACRD^†^** | **BMI** | -0.77 | (-1.53 | -0.01) | 0.000 | 0.40 | (-1.22 | 2.03) | 0.000 | -0.83 | (-1.91 | 0.24) | 0.000 |  |
| ***d*** | **G3 Tox*** | **BMI** | 1.02 | (0.89 | 1.17) | 0.005 | 0.93 | (0.76 | 1.15) | 0.000 | 1.02 | (0.88 | 1.17) | 0.000 |  |
| ***b*** | **OS^‡^** | **ARDI** | 1.04 | (0.99 | 1.10) | 0.000 | 1.04 | (0.91 | 1.18) | 0.000 | 1.00 | (0.94 | 1.07) | 0.000 |  |
|  | **DFS^‡^** | **ARDI** | 1.00 | (0.97 | 1.04) | 0.000 | 1.09 | (1.00 | 1.19) | 0.000 | 1.02 | (0.97 | 1.07) | 0.000 |  |
|  | **CSS^‡^** | **ARDI** | 1.00 | (0.93 | 1.07) | 0.000 | 1.09 | (0.90 | 1.32) | 0.000 | 1.02 | (0.95 | 1.11) | 0.000 |  |
|  | **OS^‡^** | **ACRD** | 0.92 | (0.90 | 0.95) | 0.000 | 1.01 | (0.93 | 1.09) | 0.003 | 0.99 | (0.92 | 1.08) | 0.005 |  |
|  | **DFS^‡^** | **ACRD** | 0.95 | (0.93 | 0.98) | 0.000 | 1.03 | (0.95 | 1.11) | 0.003 | 1.00 | (0.98 | 1.02) | 0.000 |  |
|  | **CSS^‡^** | **ACRD** | 0.93 | (0.90 | 0.96) | 0.000 | 1.07 | (0.97 | 1.17) | 0.000 | 0.99 | (0.91 | 1.08) | 0.005 |  |
| ***e*** | **ARDI** | **G3+ Tox** | -4.03 | (-6.47 | -1.58) | 4.238 | -0.31 | (-2.26 | 1.64) | 0.128 | -0.12 | (-1.55 | 1.31) | 0.000 |  |
|  | **ACRD** | **G3+ Tox** | -9.49 | (-12.57 | -6.40) | 4.148 | -2.83 | (-6.84 | 1.18) | 0.000 | -1.34 | (-5.38 | 2.70) | 7.398 |  |
| ***f*** | **OS^‡^** | **G3+ Tox** | 1.40 | (1.01 | 1.95) | 0.000 | 1.66 | (0.83 | 3.36) | 0.000 | 0.89 | (0.61 | 1.31) | 0.000 |  |
|  | **DFS^‡^** | **G3+ Tox** | 1.19 | (0.94 | 1.50) | 0.000 | 1.18 | (0.72 | 1.91) | 0.000 | 1.00 | (0.76 | 1.31) | 0.000 |  |
|  | **CSS^‡^** | **G3+ Tox** | 1.06 | (0.69 | 1.62) | 0.000 | 1.10 | (0.38 | 3.23) | 0.000 | 1.09 | (0.67 | 1.75) | 0.000 |  |
| ***c*** | **OS^‡^** | **BMI** | 1.00 | (0.87 | 1.15) | 0.000 | 0.86 | (0.54 | 1.39) | 0.085 | 0.97 | (0.83 | 1.14) | 0.000 |  |
|  | **DFS^‡^** | **BMI** | 0.90 | (0.72 | 1.14) | 0.038 | 0.90 | (0.71 | 1.13) | 0.000 | 1.05 | (0.94 | 1.17) | 0.000 |  |
|  | **CSS^‡^** | **BMI** | 0.95 | (0.79 | 1.14) | 0.000 | 1.05 | (0.61 | 1.78) | 0.052 | 0.98 | (0.80 | 1.21) | 0.000 |  |

SI19 | Path *a* total effect sensitivity analysis

Summary effect estimates for path *a* sensitivity analyses. **S0** Main analysis (for comparison); **S1** Excluding 3 months chemotherapy; **S2** Excluding capecitabine regimens; **S3** Excluding deaths within 6 months; **S4** Excluding potential data errors; **S5** Complete case TOX1; **S6** Complete case TOX2; **S7** Excluding small trials; **S8** Conservative confidence intervals calculated using the Hartung Knapp Siddik Jonkman method (HKSJ).

SI20 | Path *d* total effect sensitivity analysis

Summary effect estimates for path *d* sensitivity analyses. **S0** Main analysis (for comparison); **S1** Excluding 3 months chemotherapy; **S2** Excluding capecitabine regimens; **S3** Excluding deaths within 6 months; **S4** Excluding potential data errors; **S5** Complete case TOX1; **S6** Complete case TOX2; **S7** Excluding small trials; **S8** Conservative confidence intervals calculated using the Hartung Knapp Siddik Jonkman method (HKSJ).

Total

SI21 | Path *b* total effect sensitivity analysis (overall survival)

Summary effect estimates for path *b* (overall survival) sensitivity analyses. **S0** Main analysis (for comparison); **S1** Excluding 3 months chemotherapy; **S2** Excluding capecitabine regimens; **S3** Excluding deaths within 6 months; **S4** Excluding potential data errors; **S5** Complete case TOX1; **S6** Complete case TOX2; **S7** Excluding small trials; **S8** Conservative confidence intervals calculated using the Hartung Knapp Siddik Jonkman method (HKSJ).

SI22 | Path *e* total effect sensitivity analysis

Summary effect estimates for path *e* sensitivity analyses. **S0** Main analysis (for comparison); **S1** Excluding 3 months chemotherapy; **S2** Excluding capecitabine regimens; **S3** Excluding deaths within 6 months; **S4** Excluding potential data errors; **S5** Complete case TOX1; **S6** Complete case TOX2; **S7** Excluding small trials; **S8** Conservative confidence intervals calculated using the Hartung Knapp Siddik Jonkman method (HKSJ).

SI23 | Path *f* total and direct effects sensitivity analysis (overall survival)

Summary effect estimates for path *f* (overall survival) sensitivity analyses. **S0** Main analysis (for comparison); **S1** Excluding 3 months chemotherapy; **S2** Excluding capecitabine regimens; **S3** Excluding deaths within 6 months; **S4** Excluding potential data errors; **S5** Complete case TOX1; **S6** Complete case TOX2; **S7** Excluding small trials; **S8** Conservative confidence intervals calculated using the Hartung Knapp Siddik Jonkman method (HKSJ).

SI24 | Path *c* total and direct effects sensitivity analysis (overall survival)

Summary effect estimates for path c (overall survival) sensitivity analyses. **S0** Main analysis (for comparison); **S1** Excluding 3 months chemotherapy; **S2** Excluding capecitabine regimens; **S3** Excluding deaths within 6 months; **S4** Excluding potential data errors; **S5** Complete case TOX1; **S6** Complete case TOX2; **S7** Excluding small trials; **S8** Conservative confidence intervals calculated using the Hartung Knapp Siddik Jonkman method (HKSJ).
